# The Emerging Role of Multimodal Artificial Intelligence in Urological Surgery

**DOI:** 10.3390/curroncol32120665

**Published:** 2025-11-27

**Authors:** Leonhard Buck, Jakob Kohler, Julian Risch, Reha-Baris Incesu, Konrad Hügelmann, Marie-Luise Weiss, Oscar Weische, Patricia Schließer, Hans Christoph von Knobloch, Niclas C. Blessin, Thorsten Bach, Jonas Jarczyk, Philipp Nuhn, Severin Rodler

**Affiliations:** 1 Department of Urology, University Hospital Schleswig-Holstein Campus Kiel, 24105 Kiel, Germany; jakob.kohler@uksh.de (J.K.);; 2 Kurt-Semm Center for Laparoscopic and Robotic Surgery, University Hospital Schleswig-Holstein Campus Kiel, 24105 Kiel, Germany; 3 Department of Pathology, University Hospital Schleswig-Holstein Campus Kiel, 24105 Kiel, Germany; 4 Working Group on Artificial Intelligence and Digitalization of the German Society of Urology

**Keywords:** urologic oncology, multimodal artificial intelligence, generative AI, large language models, surgical robotics, perioperative care, ethical challenges

## Abstract

Background: Multimodal artificial intelligence (MMAI) is transforming urological oncology by enabling the seamless integration of diverse data sources, including imaging, clinical records and robotic telemetry to facilitate patient-specific decision-making. Methods: This narrative review summarizes the current developments, applications, opportunities and risks of multimodal AI systems throughout the entire perioperative process in uro-oncologic surgery. Results: MMAI demonstrates quantifiable benefits across the entire perioperative pathway. Preoperatively, it improves diagnostics and surgical planning via multimodal data fusion. Intraoperatively, AI-assisted systems provide real-time context-based decision support, risk prediction and skill assessment within the operating theater. Postoperatively, MMAI facilitates automated documentation, early complication detection and personalized follow-up. Generative AI further revolutionizes surgical training through adaptive feedback and simulations. However, critical limitations must be addressed, including data bias, the barrier of closed robotic platforms, insufficient model validation, data security issues, hallucinations and ethical concerns regarding liability and transparency. Conclusions: MMAI significantly enhances the precision, efficiency and patient-centeredness of uro-oncological care. To ensure safe and widespread implementation, resolving the technical and regulatory barriers to real-time integration into robotic platforms is paramount. This must be coupled with standardized quality controls, transparent decision-making processes and responsible integration that fully preserves physician autonomy.

## Simple Summary

Artificial intelligence (AI) is transforming urological oncology surgery. While earlier systems were mainly limited to analyzing image data, modern multimodal models can combine various sources of information, such as imaging, laboratory values, robotic data and clinical documentation. This allows for more precise diagnostics, surgical planning and follow-up care. Generative AI can also enhance communication between physicians and patients, as well as support surgical training by providing realistic simulations and personalized feedback. However, there are risks associated with incomplete or distorted training data, data protection issues and unclear responsibilities in AI-supported decision-making. A better understanding of the opportunities and limitations of multimodal and generative AI is crucial to ensure its safe, ethical and clinically meaningful use in urological practice.

## 1. Introduction

The integration of artificial intelligence (AI) into surgical workflows is a major challenge, but it is a proposed cornerstone of future development in this exciting field of uro-oncology surgery [1]. In the early stages of surgical AI research, most applications were single-modality and single-task systems, typically based on computer vision models for step and instrument recognition or complication detection from intraoperative videos [2]. However, these models are limited to visual signals and do not consider the broader clinical context.

In urology, however, extensive data are generated before, during and after surgery, including laboratory data, imaging data, electronic health record (EHR) data, robotic telemetry and surgical videos [3,4]. This data is typically neither structured nor systematically recorded. By combining these heterogeneous data sources with large language models (LLMs), it is now possible to train multimodal models that can not only detect patterns but also explain them in clinically meaningful ways [5]. This enables richer context generation, automated reporting and training or feedback systems. The potential of this paradigm is particularly pronounced in uro-oncology, where procedures such as robotic-assisted radical prostatectomy (RARP), radical cystectomy (RC) and partial nephrectomy (PN) are highly standardized.

In this context, it is important to distinguish between the different concepts of AI.

“Single-modality AI” uses one type of data, such as endoscopic video, CT scans or pathology slides, to perform a specific task, such as segmentation, detection or prediction.

“Integrated AI” involves incorporating one or more AI tools into a clinical workflow or device (e.g., smart overlays on a robotic console) but does not necessarily combine different data modalities within one model.

In contrast, multimodal artificial intelligence (MMAI) explicitly combines two or more different types of data, such as imaging, clinical variables and digital pathology, to learn joint representations across these inputs [6]. Therefore, MMAI has the capacity to support end-to-end, patient-specific decision-making across the perioperative pathway, rather than optimizing isolated tasks.

This non-systematic narrative review aims to provide an overview of the opportunities and risks of multimodal concepts using MMAI in uro-oncological surgery across the preoperative, intraoperative and postoperative phases, as well as in surgical training.

### Literature Search and Selection

This narrative review is based on a non-systematic literature search conducted in PubMed and Google Scholar up to October 2025. The search terms used were, among others, “multimodal artificial intelligence”, “artificial intelligence”, “urology”, “urologic surgery” and “robot-assisted surgery”.

Relevant peer-reviewed studies, clinical trials, feasibility studies and technical reports relating to the use of multimodal AI in the preoperative, intraoperative and postoperative phases of urological surgery were included. Non-peer-reviewed preprints and papers lacking methodological detail were excluded. This selection aims to reflect the breadth of available evidence, rather than claiming to be exhaustive.

## 2. Technological Background

**Figure 1:**
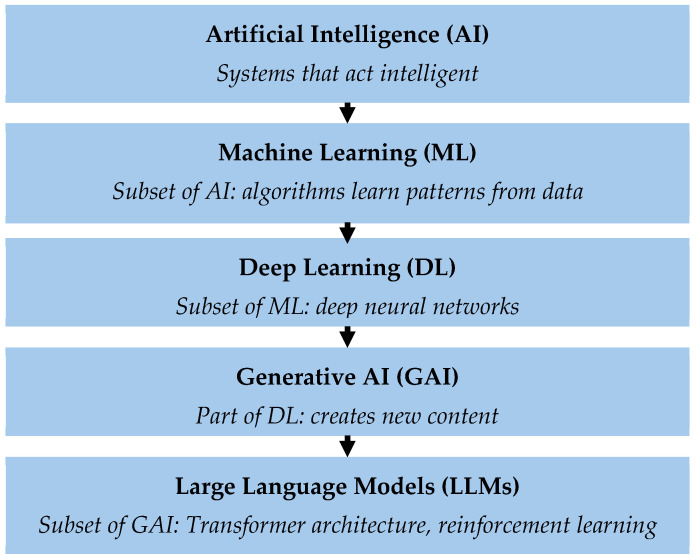
Hierarchical representation of the different levels of artificial intelligence (AI). Artificial Intelligence (AI) describes systems that can perform tasks with human-like intelligence. Machine learning (ML) is a subset of AI in which algorithms learn independently from data and recognize patterns. Deep learning (DL) uses multi-layered neural networks to understand complex relationships. Generative AI (GAI) is a subset of deep learning that can generate new content, such as text, images or simulations. Large language models (LLMs) are specialized GAI models designed for language processing. The Transformer architecture enables AI models to recognize relationships between words in a sentence by analyzing their meaning in the context of the entire text. This enables the model to understand language and respond meaningfully. LLMs are further trained using “Reinforcement Learning with Human Feedback” (RLHF) to generate more precise and understandable answers.

To gain a better understanding of current developments in urological surgery, it is necessary to provide an overview of the fundamental principles of AI, particularly generative AI. These relationships are depicted schematically in Figure 1.

The term “artificial intelligence” (AI) encompasses various technologies that enable machines to perform tasks that normally require human intelligence [7]. Machine learning (ML) is a subgroup of AI that uses statistical methods to learn from data, recognize patterns and make predictions [8]. As a further development of ML, deep learning (DL) uses artificial neural networks to recognize patterns from large amounts of data, like the human brain. Generative AI (GAI) is a subset of DL. GAI can not only analyze and organize existing information but also create its own content, including text, images, audio files and other synthetic data. A distinction is made between natural language processing (NLP), image processing and prediction. Large language models (LLMs) are the tools for interpreting, generating and manipulating human-like text [9]. The advantage of LLMs is that they can be developed and specialized for specific tasks [10].

The quality and diversity of the underlying data are crucial for these models’ performance. In uro-oncology, for example, the data is particularly diverse. Sources of possible data include robotic and endoscopic video sequences, as well as all instrument-specific data collected intraoperatively. Perioperative data, including electronic health record (EHR) data, laboratory values, imaging data and digital pathology data are available [11]. This database enables a wide range of applications of multimodal AI (MMAI) throughout the entire perioperative process.

## 3. Perioperative Applications of MMAI in Uro-Oncological Surgery

**Figure 2:**
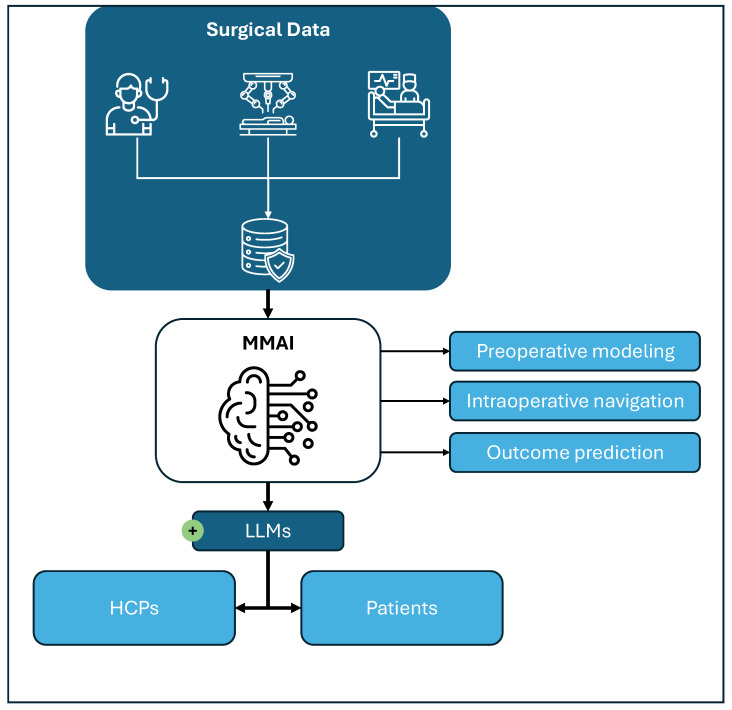
Integration of surgical data through multimodal artificial intelligence (MMAI). Surgical data from preoperative counseling and imaging, intraoperative robotic/video and monitoring data, as well as postoperative follow-up data, can be integrated into MMAI models to generate digital twins (preoperative modeling), perform intraoperative navigation and predict postoperative outcomes. Additional applications include simulation and training based on surgical data. Large language models (LLMs), as part of the MMAI, might facilitate communication and display of information generated by the MMAI to the healthcare professionals (HCPs) involved in the care of patients, as well as directly to the patient.

MMAI is envisioned to bridge the gap between single-task AI applications and the full integration of AI within clinical workflows. LLMs play a pivotal role in this context, as they can transform complex information into diverse, context-specific outputs tailored to various stakeholders, including healthcare professionals (e.g., in the operating room) and patients [5]. These outputs can be dynamically adapted to situational requirements, such as the desired level of empathy, depth of information and mode of visualization. As MMAI systems are not yet implemented in clinical practice, this work focuses on current challenges and early technological applications across the preoperative, intraoperative and postoperative settings, as well as in surgical training (see Figure 2).

### 3.1. Preoperative

Before planning uro-oncological surgery, the treating urologist must take several preparatory steps. First, they assess the patient’s medical and treatment history, followed by a review of imaging modalities, current guidelines and the available literature [5]. Therefore, there are multiple data sources that must be evaluated together to guide surgical decision-making.

In urological oncology, several conventional imaging techniques are routinely used. These include ultrasonography, computed tomography (CT), magnetic resonance imaging (MRI), skeletal scintigraphy and positron emission tomography (PET) [12]. Conventional imaging techniques are increasingly being supplemented by molecular imaging, such as the use of prostate-specific membrane antigen (PSMA) tracers. These techniques allow tumors and lymph nodes to be localized more precisely and can be used with robotic systems during surgery [13,14,15]. The importance of multiparametric MRI (mpMRI) has also increased, particularly in the diagnosis and preoperative evaluation of prostate cancer [16].

Various imaging modalities can be integrated through MMAI. These systems combine imaging data with clinical parameters and genomic markers, where applicable, to improve surgical planning and decision-making [17].

Artificial intelligence can complement imaging-based and anatomical planning by contributing to risk assessment and prognostication in uro-oncology. AI models can categorize the risk of progression in patients with intermediate-risk prostate cancer based on digitized histopathology [18]. AI-enabled prognostic models predict the risk of metastatic progression using whole-slide images of core biopsies or prostatectomy specimens. In a retrospective validation cohort of 176 patients, this deep learning model stratified intermediate-risk patients with high precision, achieving a hazard ratio (HR) of 4.66 (*p* < 0.001) for metastasis-free survival and an HR of 4.35 for biochemical recurrence-free survival compared to low-risk groups. This suggests that AI-derived histopathological biomarkers could complement genomic and clinical models for personalized therapy planning [19]. AI-assisted histopathological grading systems have demonstrated the ability to detect, grade and quantify prostate cancer with high accuracy. These systems achieve near-perfect agreement with expert pathologists and reduce interobserver variability [20]. These tools can improve diagnostic consistency, refine prognostic assessment and enhance personalized surgical decision-making. For instance, multi-classifier systems that combine long non-coding RNA (lncRNA)-based genomic classifiers, deep learning histology models and clinicopathological data have been shown to be more accurate than any single classifier at predicting recurrence-free survival in papillary renal cell carcinoma [21]. MMAI synthesizes diverse data types into clinically actionable insights, enabling more accurate and individualized prognostication and risk assessment in urologic oncology [22,23].

In recent years, three-dimensional (3D) reconstructions have become an integral part of the preoperative planning process in urological surgery. AI-based 3D reconstructions and virtual simulations facilitate patient-specific surgical planning and improve visualization of intricate anatomical structures and their spatial relationships with tumors, blood vessels and neighboring organs [24]. This improved anatomical understanding enables more precise and safer surgical preparation. The clinical utility of such 3D models has already been demonstrated in urology, particularly when planning partial nephrectomy, radical cystectomy and robot-assisted radical prostatectomy [25].

Recent evidence further supports this development. The 3DPN trial is a multicenter randomized controlled trial (RCT) investigating the benefits of virtual interactive 3D modeling compared with conventional CT for preoperative planning in robot-assisted partial nephrectomy [26]. The ongoing trial aims to enroll 370 patients across ten centers, with total console time as the primary endpoint, serving as a surrogate for surgical efficiency. Secondary outcomes include warm ischemia time and positive surgical margin rates. This study underscores the growing importance of 3D modeling as a decision-support tool and highlights its potential to improve surgical precision and perioperative outcomes in urological oncology.

The integration of anatomical 3D kidney models, including vasculature, the pyelocaliceal system and cystic structures, can enhance tumor localization and enable safer hilar dissection during robot-assisted partial nephrectomy. These models may be displayed on auxiliary monitors, integrated directly into robotic interfaces or superimposed using augmented reality (AR). Furthermore, the incorporation of perfusion-zone mapping allows identification of segmental arteries, facilitating selective clamping and maximizing preservation of healthy renal parenchyma [27].

Beyond renal surgery, 3D modeling has also demonstrated its value in prostate cancer surgery [28,29]. Integrating 3D-printed prostate models into nerve-sparing RARP can have a positive impact on surgical outcomes, improving intraoperative orientation and pathological communication [30].

MMAI enhances these preoperative 3D reconstructions in urological oncology further, particularly by optimizing automated segmentation, modeling and real-time intraoperative integration [31]. Using MMAI-supported algorithms, precise and automated segmentation of tumors and critical structures, such as neurovascular bundles, on preoperative MRI or CT images is possible. This facilitates the creation of high-resolution, patient-specific 3D models [17]. Furthermore, MMAI approaches can compensate the limitations of individual modalities. This increases both the precision of tumor detection and the safety of resection [32,33]. Early AI models primarily analyzed individual image modalities. However, modern multimodal systems integrate heterogeneous data sources, enabling more comprehensive and patient-specific preoperative planning.

Close interdisciplinary collaboration between radiology, urology and data science teams is essential for effectively combining these modalities and interpreting the resulting data in a clinically relevant context. The multimodal approach is a dynamic process that continuously optimizes itself through technological innovations and ongoing advances in AI [34].

MMAI can also be used not only in imaging but also preoperatively for patient education. It can provide medical information not only in text, but also in images, videos, audio and interactive formats. This enables a more personalized, understandable and engaging presentation of complex content. This has been proven to increase patient health literacy and understanding [35]. Using principles such as dual-coding theory and the reduction of cognitive overload, MMAI tools can prepare content so that it is processed both visually and verbally. This promotes the absorption and retention of relevant health information, particularly in younger or digitally savvy patient groups [36]. Initial studies show that AI-generated materials can improve the readability and accessibility of patient information, including in urology [37]. However, medical supervision is still required to ensure content accuracy and adaptation to the patient’s language level. In uro-oncology, AI has been used to translate complex information on prostate, bladder, kidney and testicular cancer into short, easy-to-understand texts for patients. In a randomized assessment, GPT-4 converted EAU guidelines into patient education materials in a mean time of 52.1 s. The AI-generated content significantly improved readability, reducing the required reading level from Flesch–Kincaid Grade 11.6 to 6.1 (*p* < 0.001), while maintaining equivalent ratings for accuracy, completeness and clarity compared to original expert-authored texts [38]. Such AI tools can support patient education and improve understanding of the disease and treatment options. Multimodal AI can also overcome barriers such as language or literacy difficulties by generating explanatory videos, infographics or spoken explanations. This is particularly relevant for patients with low health literacy or in resource-limited settings [39].

### 3.2. Intraoperative

Historically, the application of AI in surgery, and thus also in uro-oncology, was focused on image-based approaches. The focus was on computer vision, i.e., instrument and step recognition in the operating room. Intraoperative image data is automatically analyzed to identify relevant anatomical structures and support surgical steps. AI-supported systems can suggest intraoperative risk profiles, bleeding probabilities or the optimal resection line thus facilitating decision-making [17,40,41].

A key development has been the visualization and protection of delicate neurovascular structures, which are essential for functional outcomes in urologic oncology. Recent work on deep learning-based auto-contouring of prostate MRI achieved Dice similarity coefficients between 0.79 and 0.92, demonstrating robust segmentation of neurovascular bundles and periprostatic structures [42]. Accurately identifying these structures allows for nerve-sparing techniques during radical prostatectomy, which helps preserve erectile function and continence. Similarly, visualizing arterial anatomy helps minimize inadvertent vessel injury and intraoperative bleeding [31].

AI-based systems have demonstrated the ability to automatically identify and visualize critical anatomical structures, such as nerves and blood vessels during lymph node dissection. These models achieve high segmentation accuracy and near-real-time performance, suggesting their strong potential for use in surgical navigation [43]. Furthermore, deep learning models can accurately predict lymphovascular invasion from whole-slide images, demonstrating their independent prognostic value for survival prediction in bladder cancer patients [44].

Confocal laser microscopy (CLM) has enabled rapid and accurate intraoperative margin assessment for surgical margin detection during robot-assisted radical prostatectomy. CLM significantly reduced the median intraoperative assessment time to 8 min compared to 50 min for conventional frozen sections, with a high specificity of 96% for detecting positive surgical margins [45]. MMAI is a promising extension of this approach because it can automatically analyze and correlate imaging, histopathology and confocal microscopy data ex vivo on resected tissue and potentially in vivo during surgery. This integration would allow for the precise, real-time identification of positive margins without delaying the procedure.

Augmented reality (AR) systems project relevant anatomical and procedural information directly into the surgeon’s field of vision [46]. AI-driven AR navigation during partial nephrectomy can visualize the renal vasculature and its anatomical relationships intraoperatively, reducing hilar dissection time and minimizing bleeding, especially in cases of complex vascular anatomy [47]. Pure vision models have evolved further toward multimodal interaction by incorporating speech and context understanding. Recent vision-language models integrate visual perception, temporal analysis and higher-order reasoning to enable the multimodal interpretation of surgical scenes [48,49].

Therefore, MMAI applications rely on the tight integration of imaging, sensor data (e.g., tension sensors on robotic arms) and software-based decision support systems. This enables the consideration of intraoperative changes, such as organ motion, in real time, thereby increasing surgical precision [50].

In a prospective validation, the deep learning model ‘CystoNet’ achieved a sensitivity of 90.9% and a specificity of 98.6% in detecting bladder cancer during cystoscopy. The system successfully identified 95% of tumors (42 out of 44), potentially addressing the 20% miss rate associated with standard white-light cystoscopy [51]. Beyond oncology, computer vision-based navigation assistance has been successfully demonstrated in ureteroscopy, improving procedural safety and efficiency [52].

Beyond real-time visualization, deep learning models can analyze surgical video and kinematic data to assess surgeon proficiency. A multi-institutional study using RARP videos achieved 89% accuracy in identifying surgical phases and developed a parameter-based scoring system that effectively classified experts and novices with an accuracy of 86.2%. This suggests a high potential for automated, objective training and accreditation systems [53].

In the context of globalization and increasingly international surgical teams, MMAI applications can facilitate the automatic translation of intraoperative instructions and interface displays into different languages. This improves team communication and workflow [54]. The integration of these technologies is driving the evolution toward a “digital operating room,” in which all available data streams are consolidated and utilized by the surgeon, assistants and nursing staff in real time and in a context-sensitive manner.

### 3.3. Postoperative

AI can also be helpful in documentation after surgery: Surgical reports generated by LLMs can not only be created significantly faster but also better comply with guidelines. They are positively evaluated by both surgeons and patients. AI-generated, realistic image representations further improve documentation, even though human input is still required [55]. Collaboration between surgeons and AI leads to the highest levels of acceptance and quality, whereas purely AI-generated reports, while faster, are less reliable [56]. By integrating multimodal data, AI can identify postoperative complications early, create individual risk profiles and derive personalized follow-up recommendations [57]. In uro-oncology AI already enables more precise predictions of treatment success and the analysis of complex relationships between imaging, pathology and clinical parameters [58]. The automated analysis of telemetry data from robotically assisted surgeries in combination with patient data enables continuous and comprehensive monitoring of treatment outcomes. Patient inquiries and billing activities are a major cause of physician burnout [59]. In a landmark study comparing AI versus physician responses to patient queries, evaluators preferred the AI response in 78.6% of cases. The AI responses were rated significantly higher not only for quality (odds ratio 3.6) but notably also for empathy (odds ratio 9.8), suggesting a valuable role in alleviating the administrative burden of postoperative care [60]. They can also automate billing processes by extracting relevant information from surgical reports. Studies have shown them to be highly accurate in coding surgical procedures [61].

Selected studies demonstrating quantitative outcomes of AI applications in urological oncology across the perioperative course can be found in Table 1.

### 3.4. MMAI in Urological Training

Simulations are playing an increasingly important role in urologists’ training, from students to specialists. This applies not only to robotic surgery but also to endourology [62,63].

Realistic simulations are created using virtual reality (VR) and AR. These enable safe and effective surgical training. VR and AR promote technical skills and hand-eye coordination, while AI-based feedback personalizes learning and minimizes errors [64]. MMAI also enables objective analysis of learning curves and the provision of personalized feedback. This can improve surgical competence sustainably [65]. In an increasingly globalized world, surgical teams are also multinational [66]. Multimodal AI approaches combining text, images, video and speech can create language-independent learning environments. MMAI can also provide interactive case studies and adaptive learning materials tailored to the individual [67]. Multimodal generative AI can enable surgical training to take place in personalized, accessible learning environments, making training more efficient and inclusive [68].

## 4. Future Directions: Risks, Opportunities and Integration into Robotic Platforms

The integration of MMAI applications into clinical practice requires clear quality controls, data protection measures and close collaboration between healthcare professionals and AI systems to avoid misinformation and misunderstandings [69]. A primary concern involves the quality and representativeness of training data. Models relying on single-center or specialized cohorts often exhibit inherent biases, resulting in limited generalizability and the potential for false decisions in cases involving atypical anatomy or rare tumors. Furthermore, some multimodal systems utilize synthetic or simulated data, which may lack crucial clinical validity [70]. Data protection and data security are further key challenges. Multimodal systems access highly sensitive information including imaging data, electronic health records, robotic telemetry and possibly voice data. Even with pseudonymization, there is a risk of re-identification [71]. Notably, many current LLMs are not fully compliant with existing medical data protection regulations [72]. At a technical level, insufficient model fidelity and the risk of hallucinations (the generation of plausible but false information) present serious clinical risks [73]. These errors could have critical consequences in intraoperative decision-making or automated documentation. Therefore, the development of domain-specific models and continuous, rigorous validation are crucial to avoiding misinterpretations [74].

Finally, the increasing integration of AI entails socioethical and psychological risks. While AI systems optimize decision-making through objective data analysis and risk prediction, they must never replace the surgeon’s final clinical decision [75]. Physician responsibility mandates the critical evaluation of AI-generated recommendations, the consideration of individual patient factors and the integration of intuition and experience [76]. In summary, MMAI supports and complements perioperative decision-making, but the ultimate responsibility for diagnosis, treatment and explanation always rests with the attending physician.

Until now, MMAI solutions have not been fully integrated into the available robotic platforms. This direct integration has been disabled by medical device regulations as the robotic platform itself is certified [77]. Despite the technological maturity of many AI models, integrating them into commercial robotic platforms remains challenging. Currently, most robotic systems operate as ‘closed loops’ due to strict medical device regulations, which classify any software modification as a substantial change requiring re-certification. This creates an ecosystem in which third-party MMAI solutions cannot easily interface with the robotic console in real time. Furthermore, the computational latency required to process multimodal data (video, MRI and telemetry) poses a safety risk during live surgery. Future platforms must therefore adopt open application programming interface (API) architectures that allow for secure, low-latency edge computing to bridge the gap between academic AI models and clinical surgical hardware.

However, new platforms are arising in urology [78]. The capability to interface with MMAI solutions developed by external providers may represent a significant strategic advantage, enabling differentiation from competitors and enhancing future surgical capabilities. In this context, the concept of an open development ecosystem for MMAI solutions, analogous to applications available on app stores, could be particularly impactful.

## 5. Conclusions

Multimodal and generative AI systems open new perspectives in urology across all phases of the surgical process: from preoperative planning and intraoperative decision-making to postoperative care and training. By combining diverse data sources, MMAI can make clinical processes more precise, efficient and personalized. At the same time, however, new challenges arise: biased training data, unclear liability, a lack of transparency and data protection risks can jeopardize patient safety. The responsible use of multimodal AI therefore requires interdisciplinary collaboration, technical validation and clear ethical guidelines. In addition, robotic platforms and their configuration might play an important role in the adoption of MMAI in urooncologic surgery. AI systems must not replace surgical decisions but rather support them. Only when humans and machines work together in a complementary manner can MMAI realize its full potential and contribute to a safe, learning and patient-centered urological metaverse.

## Figures and Tables

**Table 1:** Overview of selected studies demonstrating quantitative outcomes of AI applications in urological oncology across the perioperative course. Abbreviations: BCR (biochemical recurrence), HR (hazard ratio), LLM (large language model), N (sample size), PCa (prostate cancer), RARP (robot-assisted radical prostatectomy), RCT (randomized controlled trial), rPN (robotic partial nephrectomy), κ (Kappa coefficient).

Perioperative Phase	Study	AI/Data Modality	Key Application	Quantitative Outcome/Metrics	Study Design
Preoperative	Nair et al. [18]	Deep Learning (Digital Pathology)	Risk stratification in prostate cancer	*n* = 176; HR for BCR: 4.35 (*p* < 0.001); HR for metastasis: 4.66 (*p* < 0.001)	Retrospective
	Huang et al. [20]	Deep Learning	PCa grading (reduction of interobserver variability)	Interobserver agreement improved from 84.0% to 90.1% (*p* < 0.001); Weighted κ improved from 0.76 to 0.92	Retrospective
	Stolzenburg et al. (3DPN) [26]	Interactive 3D Modeling	Surgical planning (rPN)	Ongoing RCT (Target N = 370); Primary endpoint: Reduction of total console time	RCT (Protocol/Update)
Intraoperative	Shkolyar et al. [51]	Deep Learning	Bladder tumor detection (cystoscopy)	Sensitivity: 90.9%; Specificity: 98.6%; Detected 95% of tumors	Prospective Validation
	Baas et al. [45]	Confocal Laser Microscopy	Real-time margin assessment (RARP)	Median analysis time: 8 min vs. 50 min for frozen section; Concordance with pathology: κ = 0.80	Prospective Comparative
	Zhao et al. [53]	Deep Learning (Video/Phase Recognition)	Skill assessment (RARP)	Skill scoring system distinguished experts from novices with 86.2% accuracy	Multi-institutional Study
Postoperative	Ayers et al. [60]	Generative AI (LLM)	Patient communication and medical advice	78.6% preference for AI responses; 3.6× higher rating for quality and 9.8× higher for empathy	Cross-sectional Comparative

## Data Availability

No new data were created or analyzed in this study. Data sharing is not applicable to this article.

## Abbreviations

The following abbreviations are used in this manuscript:

AI	Artificial Intelligence
MMAI	Multimodal Artificial Intelligence
RARP	Robotic-Assisted Radical Prostatectomy
RC	Radical Cystectomy
PN	Partial Nephrectomy
ML	Machine Learning
DL	Deep Learning
GAI	Generative Artificial Intelligence
NLP	Natural Language Processing
LLM	Large Language Model
RLHF	Reinforcement Learning with Human Feedback
HER	Electronic Health Record
HCP	Healthcare Professionals
CT	Computed Tomography
MRI	Magnetic Resonance imaging
PET	Positron Emission Tomography
PSMA	Prostate-Specific Membrane Antigen
mpMRI	multiparametric MRI
lncRNA	long non-coding RNA
3D	Three-Dimensional
AR	Augmented Reality
CLM	Confocal Laser Microscopy
VR	Virtual Reality
API	Application Programming Interface
